# Prosthetic Rehabilitation of a Nasomaxillary Defect Utilizing a two-Component Prosthesis: a Clinical Report

**DOI:** 10.30476/DENTJODS.2019.77866.

**Published:** 2020-09

**Authors:** Mahesh Gandhewar, Tejaswini A Bankar, Audrey Selecman, Swati Ahuja

**Affiliations:** 1 Dept. of Prosthodontics, ACPM Dental College, Dhule, India; 2 Resident, Dept. of Prosthodontics, ACPM Dental College, Dhule, India; 3 Dept. of Prosthodontics, University of Tennessee Health Science Center, Memphis, India

**Keywords:** Obturator, Nasomaxillary defect, Prosthetic rehabilitation

## Abstract

Nasomaxillary defects may affect patients’ speech, mastication, swallowing, breathing, quality of life, psychology, and social behavior. A combination of surgical reconstruction and prosthetic rehabilitation is frequently required to restore optimal function and esthetics. Of particular concern are the size, weight and contour of the prosthesis, as they can drastically affect comfort, retention, masticatory function and ease of insertion and removal. This clinical case report describes the prosthodontic rehabilitation of a partially edentulous patient with a nasomaxillary defect (Aramany class VI) with a two-component prosthesis joined by magnets.

## Introduction

A nasomaxillary defect is the confluence of the oral and nasal cavity as the result of trauma, burns, infection, and surgical removal of tumors or congenital abnormalities [ 1 - 4 ]. These defects may affect speech, mastication, swallowing, breathing, quality of life, psychology, and social behavior [ 5 - 8 ]. Obturation of the defect prevents escape of air, fluid and food between the nasal and oral cavities and is rarely accomplished by surgical reconstruction alone [ 4 - 8 ]. Optimal function and esthetics often requires prosthesis for replacement of occlusal, alveolar, palatal, nasal and orbital structures [ 9 , 13 ]. Increasing the size, weight and contour of the prosthesis can drastically affect comfort, retention, masticatory function, and ease of insertion and removal [ 14 ].

A large obturator prosthesis utilizing an attachment system anchored by implants improves the stress distribution, masticatory function, and oral health related quality of life (OHRQOL) [ 15 ]. However, the use of implants may be precluded in patients with limited finances, poor health, and available bone. Optimizing broad stress distribution on the remaining soft tissues and teeth can be compromised by a path of insertion dictated by the contours of the nasal bulb. Therefore, larger defects may benefit from a detachable two-component prosthesis that engages nasal cavity undercuts otherwise unavailable if the path of insertion was dictated by the abutment teeth [ 4 ]. This clinical case report describes the prosthodontic rehabilitation of a partially edentulous patient with an Aramany class VI nasomaxillary defect [ 16 ] with a novel two-component prosthesis joined together by magnets after insertion.

## Case Presentation

**Figure 1:**
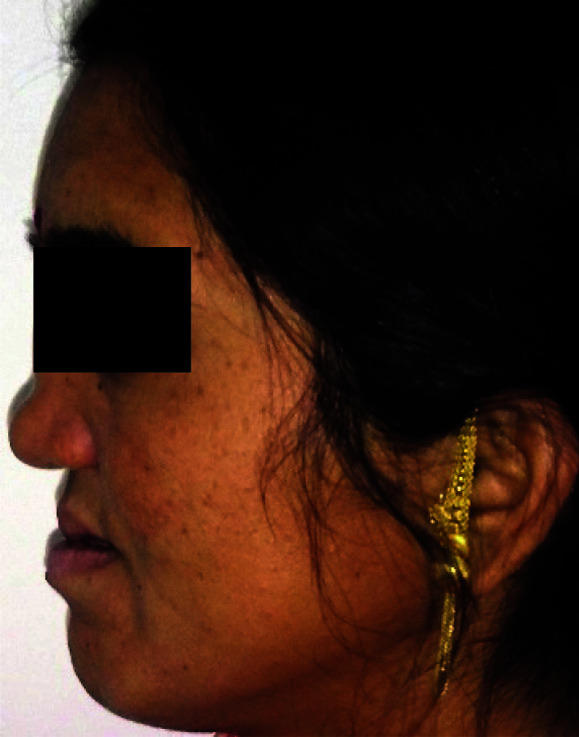
Profile view of the patient.

**Figure 2:**
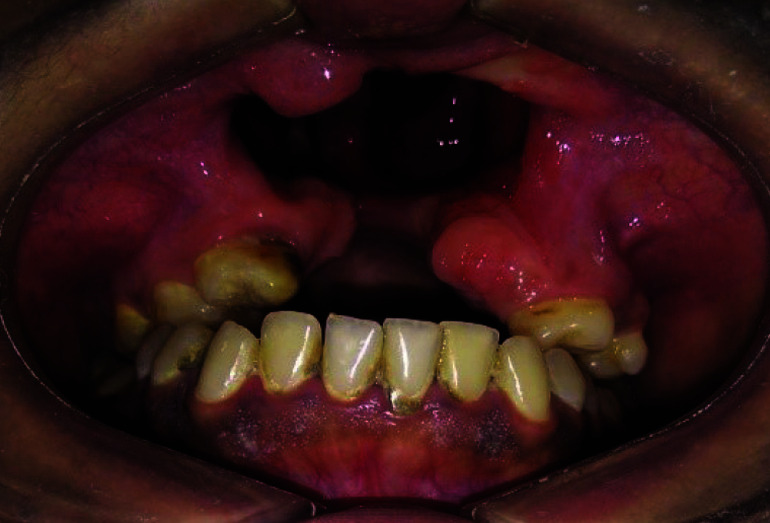
Intraoral view of the defect.

A35-year-old female patient reported to the department of prosthodontics at the A.C.P.M. Dental College, Dhule for replacement of her existing prosthesis. The extraoral exam revealed a midfacial deficiency and loss of nasal prominence (Figure 1). An intraoral examination revealed an Aramany class VI nasomaxillary defect (anterior defect with remaining posterior teeth). Missing teeth included #5-12 and #14 (Figure 2). The defect was the result of post-trauma surgical resection of the nasal and premaxillary bone. Her existing acrylic removable partial dental prosthesis exhibited poor functional mechanics and failed to obturate the defect. 

The patient desired a new prosthesis that would provide optimum function, comfort, and esthetics without surgical intervention. Considering the size of the defect, the design necessitated an innovative approach to restore facial harmony, permit nasal breathing, obturate the oronasal communication, replace missing teeth, and restore function. A novel two-component design satisfied the requisites of the prosthesis with a simple path of insertion.

**Figure 3:**
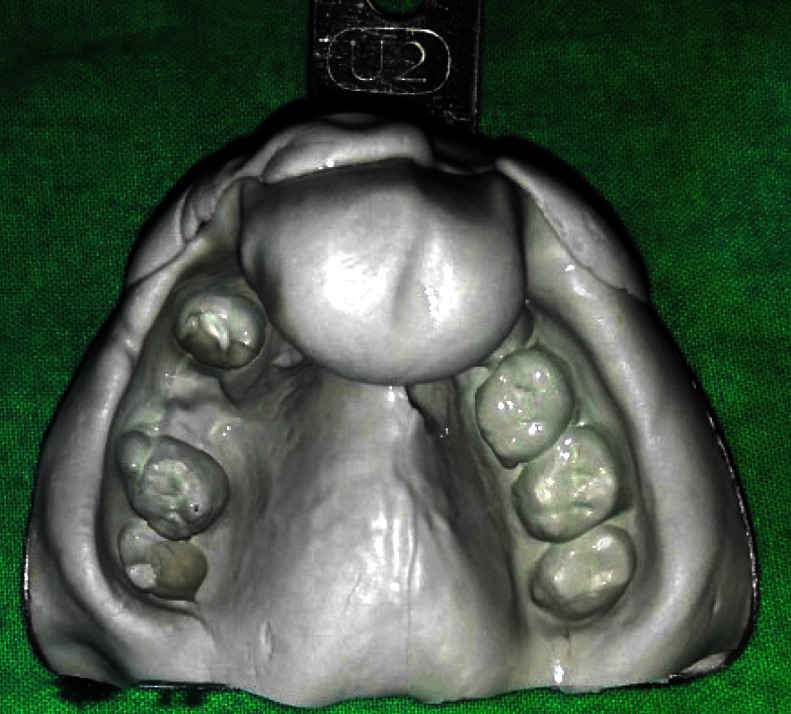
Diagnostic maxillary impression.

**Figure 4:**
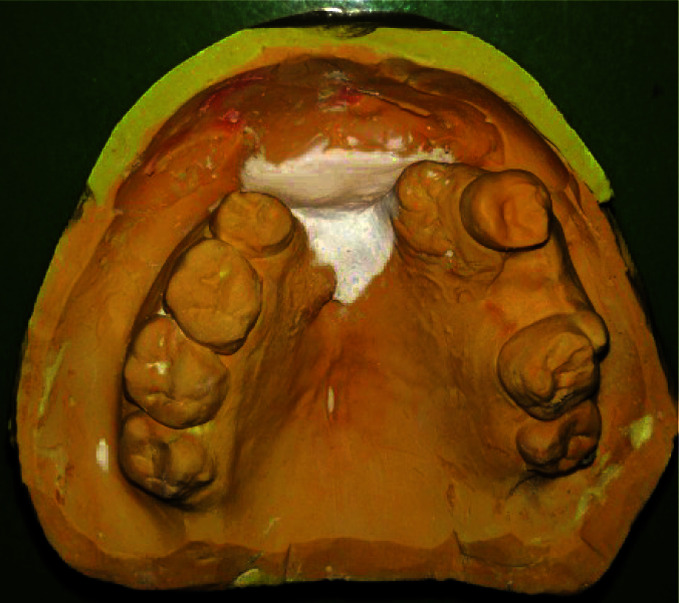
Defect undercut blocked with dental stone.

Diagnostic and definitive maxillary and mandibular impressions were made in alginate (Algitex, DPI) within prefabricated plastic trays (Figure 3). Patency of the airway was confirmed by reinserting the maxillary impression into the patient’s oral cavity and asking if she could breathe properly through her nose. A fogged mouth mirror verified air was expelled through the nasal cavities. The impressions were poured in type III dental stone (Vinayak Gypsum and interiors Pvt Ltd.) Non-viable undercuts within the defect on the maxillary cast were blocked with type III dental stone (Figure 4). A separating agent (Cold mould seal, Pyrax) was applied to the cast. 

**Figure 5:**
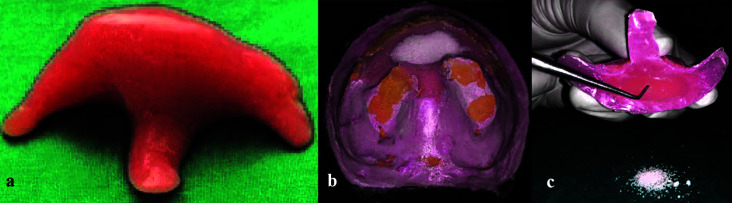
a: Nasal component, b: Salt incorporated between doughy acrylic resin layers, c: Removal of salt particles.

**Figure 6:**
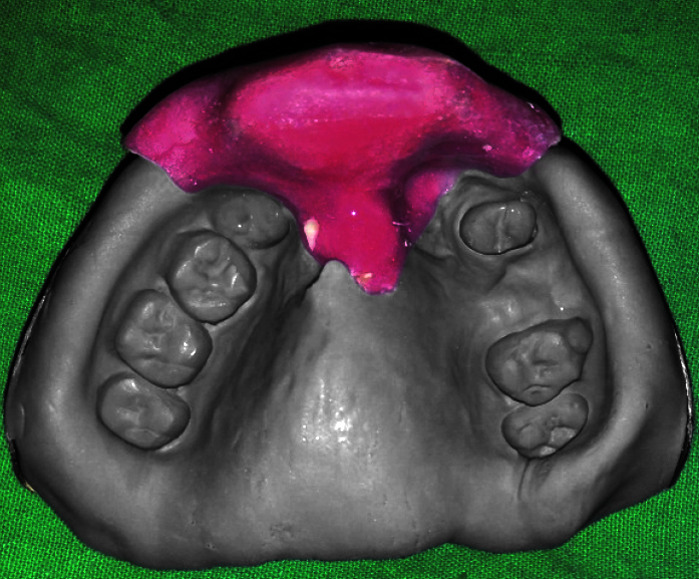
Master impression with nasal component in position.

**Figure 7:**
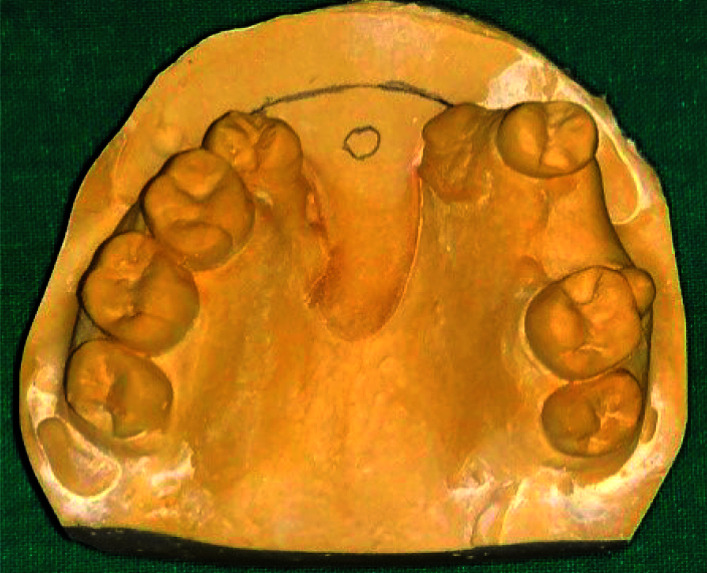
Master cast (after removal of the nasal component).

**Figure 8:**
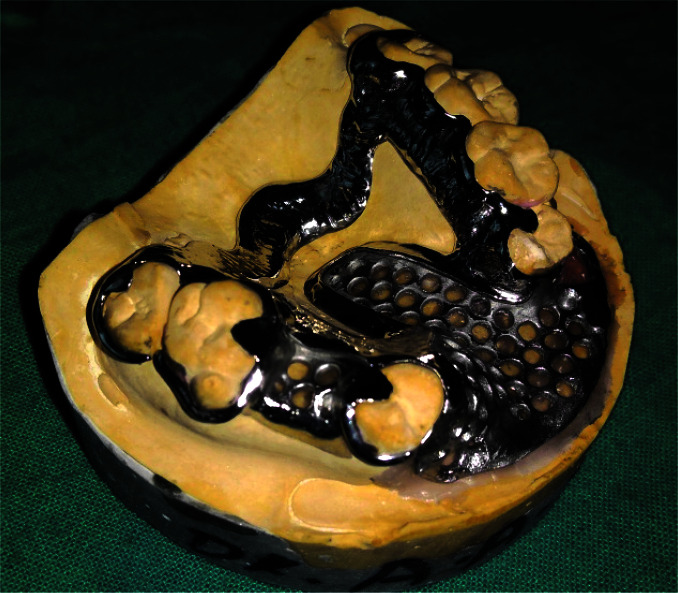
Cast partial denture framework.

Auto polymerizing acrylic resin (DPI-RR Cold Cure TM, DPI, India) was mixed per the manufacturer’s instructions and adapted to the cast’s defect to fabricate a trial nasal obturator (Figure 5a). The obturator was evaluated intraorally and adjusted using slow speed rotary instruments, as necessary. It was relined with a soft lining material (Permasoft, Dentsply) to improve fit, comfort and ease of insertion and removal. The final contours were duplicated in a heat-cure acrylic resin (DPI Heat CureTM, DPI, India) through compression molding procedure to improve material strength and create a smooth, solid, hygienic surface. During the packing stage of compression molding, salt particles were incorporated between the outer and the inner layers of doughy resin to fabricate a hollow obturator using the lost salt technique (Figure 5b). Post acrylization, these particles were removed through the holes made on the intaglio surface (Figure 5c) [ 17 ]. The diagnostic maxillary cast was surveyed and designed for a Kennedy Class III cast removable partial dental prosthesis (RDP). Mouth preparation was accomplished per the design requisites. With the nasal component seated in position, a definitive impression was made with silicon impression material (Zetaplus, Zhermack, BadiaPolesine (RO), Italy) (Figure 6) and the definitive cast was poured with type III dental stone (Figure 7). The definitive cast and detailed instructions were sent to a laboratory for fabrication of the cast RDP framework. The framework was then tried in the patient’s mouth and adjusted as necessary (Figure 8). A wax occlusal rim was attached to the framework and interocclusal records were registered in centric occlusion.

**Figure 9:**
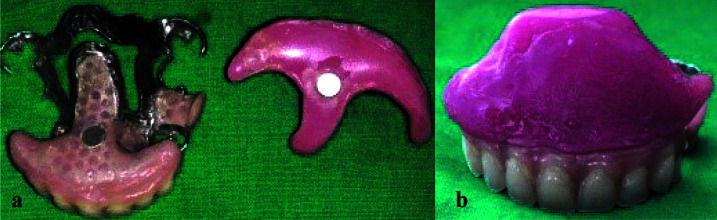
a: Magnet attached to definitive prosthesis, b: Two components prosthesis joined together by magnet.

The maxillary cast with the framework was mounted against the mandibular cast on a semi-adjustable articulator using interocclusal records. Prosthetic teeth were waxed to the framework and evaluated intraorally for phonetics, esthetics, and occlusion. The prosthetic teeth were processed to the framework and the final prosthesis was delivered to the patient. After necessary adjustments, a magnet (5mm magnet, Milestone, India) was attached to the anterior intaglio surface of the processed denture base and its counterpart to the base of the nasal obturator (Figure 9a and Figure 9b).

**Figure 10:**
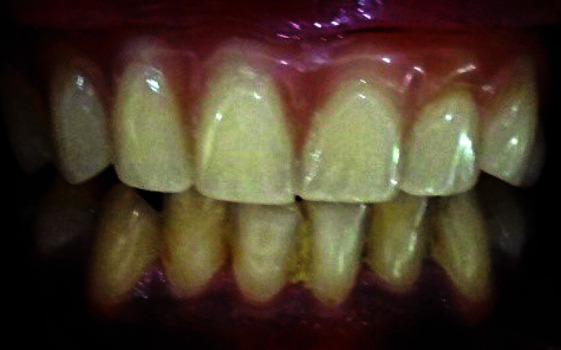
Definitive prosthesis placed in the oral cavity.

**Figure 11:**
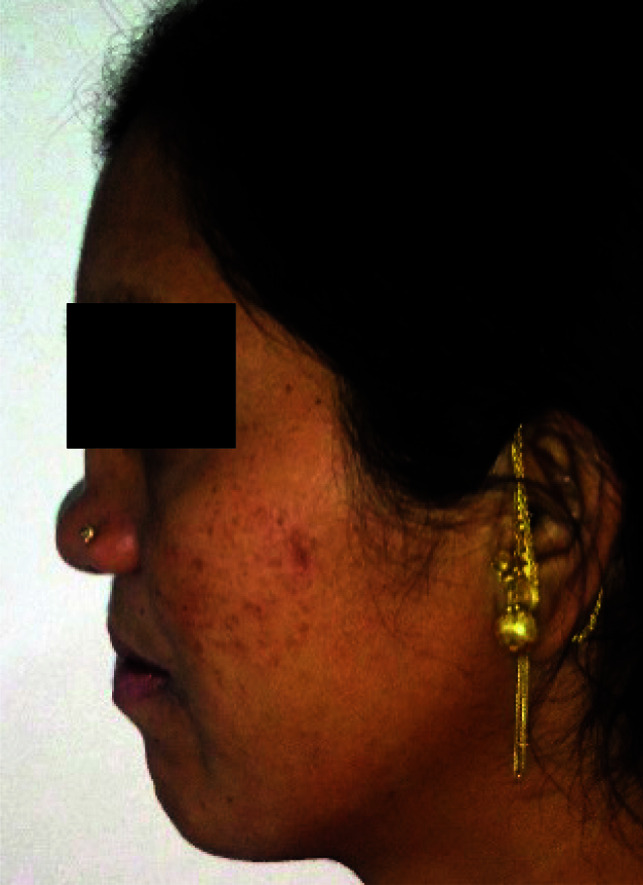
Improvement in esthetics with the definitive prosthesis.

The patient was pleased with her prosthesis (Figure 10 and 11). Home care instructions were given to patient and she was placed on regular recall and maintenance schedule.

## Discussion

Aramany class VI defects are difficult to rehabilitate due to distance of the nasal bulb from the remaining abutment teeth. The anterior segment has the propensity to cantilever into the defect without sufficient support and retention of the prosthesis. However, the path of insertion is dictated by the contours of the remaining abutment teeth.

By separating the prosthesis into two components, the nasal bulb can be fabricated for maximum obturation, support and retention without regard to the RDP. Likewise, the RDP can be fabricated for maximum support, stability and function based solely on the contours of the remaining abutment teeth and oral soft tissue. Therefore, designing each component separately maximizes mechanical design features. The magnets attach the RDP to the nasal bulb upon insertion of the RDP. Magnets allow an undefined path of insertion prior to the final connection of the component as well facilitate stability of the nasal bulb. Unfortunately, magnets do lose magnetism over time and are subject to corrosion without proper concealment. However, the cost and convenience of replacement of a magnet attachment system outweighs its limitations.

## Conclusion

Optimal rehabilitation of a patient with a nasomaxillary defect can be achieved using a two- component prosthesis attached with magnets.
